# Shoulder Arthroplasty: A Bibliometric Analysis Using the Scopus Database

**DOI:** 10.7759/cureus.61613

**Published:** 2024-06-03

**Authors:** Khairul Nizam Siron, Ren Y Kow, Nurul Ain N Md Amin, Chooi L Low, Azni N Wahid, Farahiyah Jasni, Mohd R Abidin, Siti Nor S Mustfar

**Affiliations:** 1 Department of Orthopedics, Traumatology, and Rehabilitation, International Islamic University Malaysia, Kuantan, MYS; 2 Department of Radiology, International Islamic University Malaysia, Kuantan, MYS; 3 Mechatronics Engineering Department, International Islamic University Malaysia, Gombak, MYS

**Keywords:** shoulder arthroplasty/ replacement, research trend, bibliometric analyis, anatomical hemi shoulder arthroplasty, total shoulder arthroplasty, reverse shoulder arthroplasty

## Abstract

Primary joint replacements are performed increasingly often worldwide, driven by an aging population, improvement in surgical techniques, and advancements in implant designs. While more attention has traditionally been focused on weight-bearing joints such as the hip and knee, shoulder replacement surgeries have gained increasing attention in recent years due to the population's demand for a better quality of life. Thus far, a comprehensive bibliometric analysis of shoulder arthroplasty-related publications using the Scopus database has not yet been conducted. This bibliometric analysis aims to fill this gap by reviewing the Scopus database from its inception until 2023 to examine the literature on shoulder arthroplasty. A total of 5300 publications meeting the selection criteria were included in this analysis. The turn of the century marked a significant turning point for the field of shoulder arthroplasty, with an increasing number of publications produced annually. This trend can be attributed to the improvement of implant designs, which have become more consistent and reliable over time. While the majority of articles were authored by researchers and clinicians from the United States of America (USA), publications by French authors had a higher scholarly impact in the field. There is a noticeable gap in research on shoulder arthroplasty in developing countries, possibly due to the prohibitively high cost of implants and the prioritization of other healthcare sectors. This bibliometric analysis, utilizing Scopus data, serves as a guiding light for researchers, clinicians, and policymakers, potentially fostering collaborative projects and guiding the development of future studies to further advance the field of shoulder arthroplasty, particularly in developing countries.

## Introduction and background

Thanks to advancements in technology and healthcare systems, the lifespan of humans has extended, leading to an estimated 22% of the global population being aged over 60 [1-3]. As individuals age, there is a corresponding increase in the incidence of osteoarthritis or degenerative joint disease, commonly seen in weight-bearing joints such as the hip and knee [4-8]. It is estimated that up to 240 million people are suffering from degenerative joint diseases worldwide [1-3]. Consequently, there is a projected rise in the demand for replacement surgeries such as hip and knee surgeries [3-5]. Nevertheless, degenerative disease of synovial joints can also affect non-weight-bearing joints such as the shoulder (glenohumeral) joint [1,2]. Caucasian populations, female patients, individuals with increasing weight, and those who engaged in manual labor are at a higher risk of developing shoulder osteoarthritis [2]. In fact, it is the most common cause of shoulder pain in elderly patients, leading to loss of shoulder function, depression, and reduced quality of life [2].

Since Neer's groundbreaking development of the first anatomic shoulder replacement using a vitallium prosthesis in the 1950s, the technology of shoulder replacement has advanced steadily [2,9-12]. This progress has rendered it a viable option for treating patients with severe shoulder osteoarthritis. Currently, hemiarthroplasty and total shoulder arthroplasty (both anatomic and reverse) have demonstrated effectiveness in alleviating pain and enhancing shoulder function among patients with severe shoulder arthritis [9-12]. In 2017, it was estimated that over 800,000 patients were living in the United States with shoulder replacements, and this figure is anticipated to rise owing to the increasing life expectancy of the global population [9,10]. Reports on the incidence of shoulder osteoarthritis are scarce in the literature, but it is estimated that 16% of patients aged 65 and above have radiographic evidence of shoulder osteoarthritis [1,9-12]. 

Recently, data mining has significantly facilitated the review process in research. Data collected from platforms such as Google Trends can provide valuable insights into the interests of the population and aid in making informed predictions [13,14]. Similarly, the availability of software and databases has streamlined bibliometric analysis [15-18]. Bibliometric analysis serves multifaceted purposes within a research field. It not only summarizes research interests but also evaluates academic quality and impact through metrics such as publication counts and citations [16-18]. Additionally, it measures relationships and clustering within the field, unveiling collaboration between authors, institutions, and countries. Moreover, bibliometric analysis aids in identifying research gaps, providing valuable insights for further research and advancement within the field [17-20]. While bibliometric analyses of shoulder arthroplasty have been conducted, these analyses have relied solely on the Web of Science Core Collection database for review [19,20]. Our review aims to perform a comprehensive bibliometric analysis of the literature on shoulder arthroplasty using the Scopus database.

## Review

Literature search and search strategy

**Figure 1:**
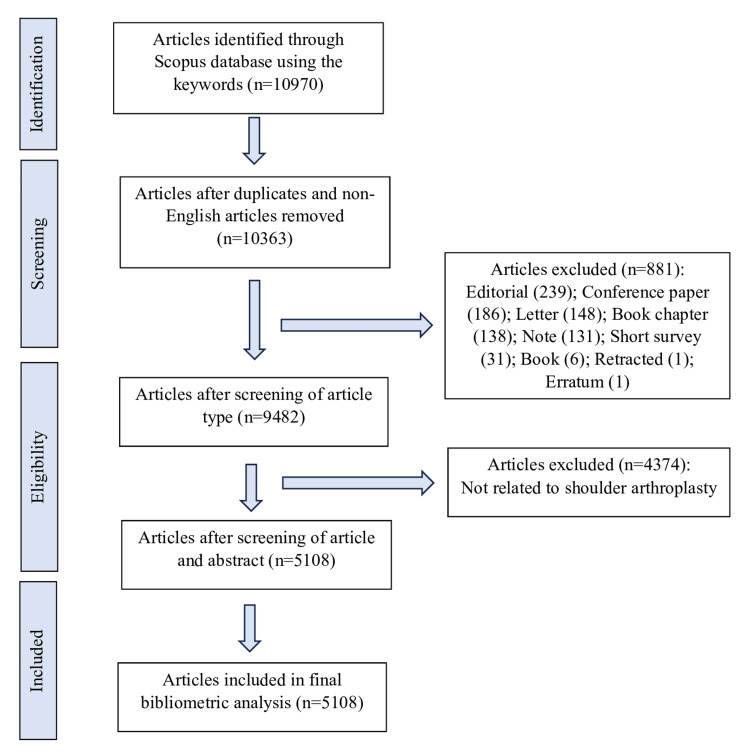
Flowchart of search results

The literature on shoulder arthroplasty was sourced from the Scopus database. The search strategy was formulated using the following keywords: (shoulder OR glenohumeral) AND (arthroplasty OR replacement). The search spanned from the inception of the database to 31 December 2023. The retrieved articles underwent screening by the two principal authors for suitability. Only English-language articles related to robotic-assisted hip and knee arthroplasty were included. Other types of publications, such as conference proceedings, book chapters, trade journals, and errata, were excluded. Arthroplasty of other joints, such as the hip, knee, elbow, hand, and ankle, were also excluded. Figure 1 illustrates the flow of the search strategy.

The retrieved data were processed using R Studio 2021 for Windows ((R Foundation, Vienna, Austria)) with the "bibliometrix" package installed in R [21]. Information such as authorship, title, publication, number of citations, affiliations, journal source, references and keywords were extracted using the R software. Data presentation, including illustrative graphs, was completed using "bibliometrix", while Microsoft Excel 2019 (Microsoft, Redmond, Washington) was employed for data organization.

Results

Annual Scientific Production and Journal

**Figure 2:**
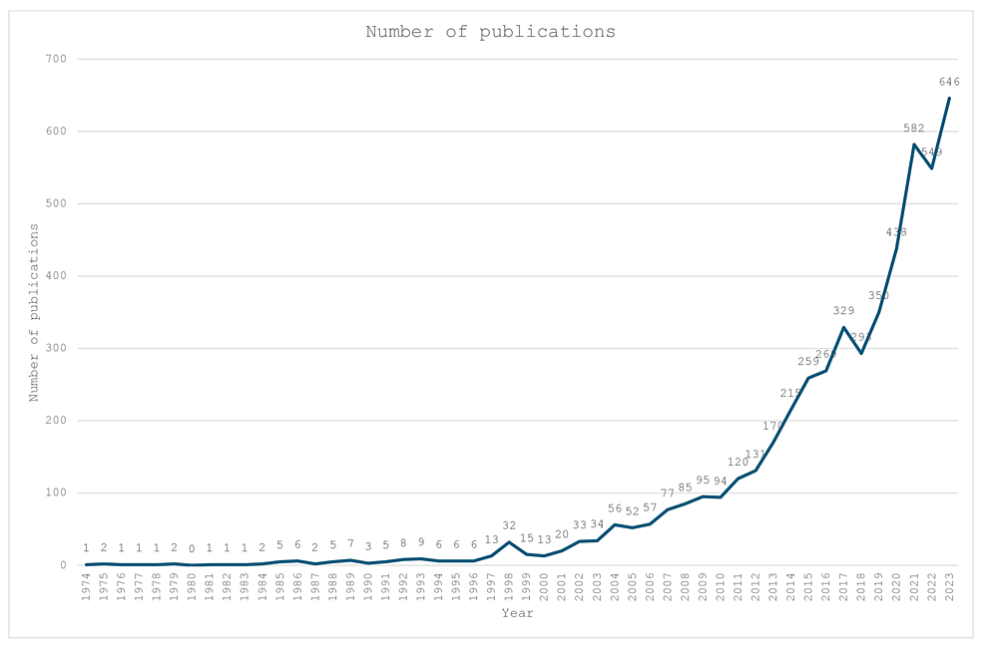
Number of publications over the years Figure made by authors

A total of 10,970 articles were retrieved from the Scopus database using the keywords presented. After screening by the lead authors, only 5108 articles met the inclusion and exclusion criteria, and they were included in this review. As shown in Figure 2, the first article related to this topic was published in 1974, and the number of annual publications remained low until 2011, where it started to increase slowly. The number of annual publications increased exponentially from 2017 onwards, reaching a peak of 646 publications per year in 2023.

All the 5108 articles were published in various journals. Table 1 summarises the top 10 journals with the highest number of publications in the field of shoulder arthroplasty. The Journal of Shoulder and Elbow Surgery (JSES) leads the table with 1445 articles. This was followed by Seminars in Arthroplasty JSES (410 articles), Shoulder and Elbow (171 articles), JSES International (165 articles), International Orthopaedics (142 articles), Journal of Bone and Joint Surgery (132 articles), Clinical Orthopaedics and Related Research (112 articles), Orthopedics (109 articles), Archives of Orthopaedic and Trauma Surgery (96 articles), and Orthopaedics and Traumatology: Surgery and Research (80 articles). Unsurprisingly, the four top journals with the highest number of publications are primarily dedicated to shoulder and elbow research.

**Table 1:** Top 10 journals with the highest number of publications JSES - Journal of Shoulder and Elbow Surgery

Journal	Number of publications	Percentage
Journal of Shoulder and Elbow Surgery	1445	28.3
Seminars in Arthroplasty JSES	410	8.0
Shoulder and Elbow	171	3.3
JSES International	165	3.2
International Orthopaedics	142	2.8
Journal of Bone and Joint Surgery	132	2.6
Clinical Orthopaedics and Related Research	112	2.2
Orthopedics	109	2.1
Archives of Orthopaedic and Trauma Surgery	96	1.9
Orthopaedics and Traumatology: Surgery and Research	80	1.6

Authors And Their Affiliations

Table 2 presents the top 10 authors with the highest number of publications in the field of shoulder arthroplasty. Among all the authors, Sperling JW published the highest number of articles, totaling 186 articles. This was followed by Wright TW (144 articles), Walch G (139 articles), Cofield RH (135 articles), Zuckerman JD (126 articles), Namdari S (93 articles), Boileau P (92 articles), Romeo AA (90 articles) Denard PJ (88 articles), and Dines DM (88 articles).

**Table 2:** Top 10 authors with the highest number of publications

Institution	Number of articles	Percentage
Sperling JW	186	3.6
Wright TW	144	2.8
Walch G	139	2.7
Cofield RH	135	2.6
Zuckerman JD	126	2.5
Namdari S	93	1.8
Boileau P	92	1.8
Romeo AA	90	1.7
Denard PJ	88	1.7
Dines DM	88	1.7

Table 3 summarizes the top 10 institutions with the highest number of publications in the field of shoulder arthroplasty. Authors from Mayo Clinic contributed the highest number of publications, with a total of 847 publications. This was followed by Hospital for Special Surgery (501 publications), University of Florida (496 publications), Rush University Medical Center (435 publications), Icahn School of Medicine at Mount Sinai (245 publications), Balgrist University Hospital (202 publications), University of California (199 publications), Medical University of South Carolina (190 publications), University of Washington (188 publications), and Duke University Medical Center (172 publications).

**Table 3:** Top 10 institutions with the highest number of publications

Institution	Number of articles	Percentage
Mayo Clinic	847	16.6
Hospital for Special Surgery	501	9.8
University of Florida	496	9.7
Rush University Medical Center	435	8.5
Icahn School of Medicine at Mount Sinai	245	4.8
Balgrist University Hospital	202	4.0
University of California	199	3.9
Medical University of South Carolina	190	3.7
University of Washington	188	3.7
Duke University Medical Center	172	3.4

Authors' Country and International Collaboration

Table 4 shows the top 10 countries with the highest number of publications in the field of shoulder arthroplasty. Authors from the United States of America (USA) had the highest number of publications in this research field, totaling 2456 publications authored by researchers and clinicians from the USA. This was followed by Germany with 271 publications, the United Kingdom with 233 publications, France with 232 publications, Switzerland with 170 publications, Italy with 146 publications, Canada with 132 publications, Australia (95 publications), Korea (94 publications), and Spain with 56 publications. Among the top 10 contributing countries, more than half of them (six countries) had extensive collaboration with other countries, wherein more than twenty percent of the publications were results of multi-countries collaboration. Up to 42 percent of the publications produced by authors from Switzerland were products of multinational collaboration. This was followed by France (33.3 percent), Australia (30.3 percent), Germany (29.1 percent), Canada (27.4 percent), and Italy (25.8 percent).

**Table 4:** Author's country with the highest number of publications SCP - single country production; MCP - multiple country production

Country	Number of publications	Percentage	SCP	MCP	MCP percentage
USA	2456	48.1	2201	255	10.7
Germany	271	5.3	642	70	29.1
United Kingdom	233	4.6	191	80	14.0
France	232	4.5	201	32	33.3
Switzerland	170	3.3	153	79	42.0
Italy	146	2.9	98	72	25.8
Canada	132	2.6	108	38	27.4
Australia	95	1.9	96	36	30.3
Korea	94	1.8	84	11	12.1
Spain	56	1.1	67	27	16.1

Table 5 summarizes the number of publications from collaboration between two countries. Collaboration between USA and France produced the highest number of publications, measuring at 176 publications. This was followed by France-Switzerland collaboration (62 publications), USA-Canada collaboration (57 publications), USA-Switzerland collaboration (56 publications), Germany-Switzerland collaboration (51 publications), USA-Germany collaboration (44 publications), France-Germany collaboration (41 publications), USA-Italy collaboration (30 publications), France-United Kingdom collaboration (27 publications), and Germany-Austria collaboration (25 publications).

**Table 5:** The number of publications from collaboration between two countries Only international collaborations with more than 10 publications are listed in this table

Collaboration between countries		Number of publications	Percentage
USA	France	176	3.4
France	Switzerland	62	1.2
USA	Canada	57	1.1
USA	Switzerland	56	1.1
Germany	Switzerland	51	1.0
USA	Germany	44	0.9
France	Germany	41	0.8
USA	Italy	30	0.6
France	United Kingdom	27	0.5
Germany	Austria	25	0.5
USA	United Kingdom	23	0.5
France	Australia	20	0.4
USA	Japan	18	0.4
France	Canada	17	0.3
Switzerland	Australia	17	0.3
Switzerland	Austria	16	0.3
USA	Australia	15	0.3
France	Italy	14	0.3
United Kingdom	Australia	14	0.3
United Kingdom	Italy	13	0.3
USA	Korea	13	0.3
USA	Spain	13	0.3
France	Belgium	12	0.2
USA	Belgium	12	0.2
Germany	Australia	11	0.2
USA	Austria	11	0.2
France	China	10	0.2
Germany	Canada	10	0.2
Germany	United Kingdom	10	0.2
United Kingdom	Switzerland	10	0.2

Top 10 Publications With the Highest Number of Citations

Table 6 outlines the top ten publications with the highest number of citations within the study period. The article titled "Grammont inverted total shoulder arthroplasty in the treatment of glenohumeral osteoarthritis with massive rupture of the cuff. Results of a multicentre study of 80 shoulders" received the highest number of citations with a total of 1213 citations [22]. "Treatment of painful pseudoparesis due to irreparable rotator cuff dysfunction with the Delta III reverse-ball-and-socket total shoulder prosthesis" had the second-highest number of citations (856 citations) [23]. This was followed by "Reverse total shoulder arthroplasty: a review of results according to etiology" (851 citations), "Neer Award 2005: The Grammont reverse shoulder prosthesis: results in cuff tear arthritis, fracture sequelae, and revision arthroplasty" (837 citations), "Increasing incidence of shoulder arthroplasty in the United States" (763 citations), "The reverse shoulder prosthesis for glenohumeral arthritis associated with severe rotator cuff deficiency. A minimum two-year follow-up study of sixty patients" (668 citations), "Complications of total shoulder arthroplasty" (649 citations), "Problems, complications, reoperations, and revisions in reverse total shoulder arthroplasty: a systematic review" (634 citations), "Reverse total shoulder arthroplasty. Survivorship analysis of eighty replacements followed for five to ten years" (607 citations), and "Stress shielding and bone resorption in shoulder arthroplasty" (588 citations) [24-31].

**Table 6:** Top ten publications with the highest number of citations. Mean citation - total number of citations divided by the number of years since the article was published

Number	Year	Journal	Author	Country	Title	Citation	Mean citation
1	2004	Journal of Bone and Joint Surgery (British volume)	Sirveaux et al. [22]	France	Grammont inverted total shoulder arthroplasty in the treatment of glenohumeral osteoarthritis with massive rupture of the cuff. Results of a multicentre study of 80 shoulders	1213	57.76
2	2005	Journal of Bone and Joint Surgery (American volume)	Werner et al. [23]	Switzerland	Treatment of painful pseudoparesis due to irreparable rotator cuff dysfunction with the Delta III reverse-ball-and-socket total shoulder prosthesis	856	42.80
3	2007	Journal of Bone and Joint Surgery (American volume)	Wall et al. [24]	France	Reverse total shoulder arthroplasty: a review of results according to etiology	851	47.28
4	2006	Journal of Shoulder and Elbow Surgery	Boileau et al. [25]	France	Neer Award 2005: The Grammont reverse shoulder prosthesis: results in cuff tear arthritis, fracture sequelae, and revision arthroplasty	837	44.05
5	2011	Journal of Bone and Joint Surgery (American volume)	Kim et al. [26]	USA	Increasing incidence of shoulder arthroplasty in the United States	763	54.50
6	2005	Journal of Bone and Joint Surgery (American volume)	Frankle et al. [27]	USA	The reverse shoulder prosthesis for glenohumeral arthritis associated with severe rotator cuff deficiency. A minimum two-year follow-up study of sixty patients	668	33.40
7	2006	Journal of Bone and Joint Surgery (American volume)	Bohsali et al. [28]	USA	Complications of total shoulder arthroplasty	649	34.16
8	2011	Journal of Shoulder and Elbow Surgery	Zumstein et al. [29]	France	Problems, complications, reoperations, and revisions in reverse total shoulder arthroplasty: a systematic review	634	45.29
9	2006	Journal of Bone and Joint Surgery (American volume)	Guery et al. [30]	France	Reverse total shoulder arthroplasty. Survivorship analysis of eighty replacements followed for five to ten years	607	31.95
10	2003	Journal of Shoulder and Elbow Surgery	Nagels et al. [31]	Netherlands	Stress shielding and bone resorption in shoulder arthroplasty	588	26.73

Types of Articles

Table 7 shows the breakdown of the type of shoulder arthroplasty-related articles. Upon analysis, the majority of the articles were related to reverse shoulder total arthroplasty (RTSA), with up to 37% of the articles focusing solely on RTSA. Additionally, 1.1% and 7.4% of the articles compared RTSA with hemiarthroplasty and total shoulder arthroplasty (TSA), respectively. This was followed by TSA-related articles, which accounted for 30.6%. On top of that, 2.7% compared TSA with hemiarthroplasty. Approximately 3.8% of the articles compared all three types: hemiarthroplasty, TSA, and RTSA. The remaining 11.5% of the articles did not fit into any of these categories; these mostly consisted of non-specific review articles.

**Table 7:** Types of articles TSA - total shoulder arthroplasty; RTSA - reverse total shoulder arthroplasty

Type of articles	Number of publications	Percentage
General	586	11.5
Hemiarthroplasty and TSA	136	2.7
Hemiarthroplasty and RTSA	56	1.1
Hemiarthroplasty, TSA, and RTSA	192	3.8
TSA and RTSA	381	7.4
Hemiarthroplasty	303	5.9
TSA	1562	30.6
RTSA	1892	37.0
Total	5108	100

Discussion

Developed by Elsevier, the Scopus database contains an extensive collection of peer-reviewed journals, conference proceedings, and patents from a variety of fields, such as science, technology, medicine, social sciences, arts, and humanities [15,16]. This database is appreciated for its broad scope, which encompasses research outputs from multiple disciplines, making it a valuable research resource for interdisciplinary research and collaboration. Researchers can access millions of articles and other scholarly documents, allowing them to stay ahead of the latest developments and trends in their respective fields. This database also provides citation analysis tools, making assessment of the influence and visibility of scholarly publications feasible. Metrics such as the h-index and citation counts enable researchers to gauge the significance of their work and identify influential authors and journals within their field [16]. Besides that, the Scopus database also facilitates collaboration and networking among researchers by providing authors' profile, their affiliations, and collaboration networks. Given that the Scopus database is one of the largest databases available, it is important to examine the nuances of each database, as each database has its own strengths and weaknesses. In this bibliometric analysis, we delve into the analysis of shoulder arthroplasty using the Scopus database. 

Upon reviewing all the shoulder arthroplasty-related publications, the number of publications began to increase at the turn of the century. Nevertheless, growth remained slow until 2010, when this field experienced exponential growth. This may be attributed to advancements in reverse shoulder arthroplasty implant design. In the 1990s, the reverse shoulder arthroplasty system introduced by Paul Grammont focused on four key principles: 1) stability of the implant; 2) convex weight-bearing part and concave supporting part; 3) centering of the glenoid sphere at or within the glenoid neck; and 4) medialization and distalization of the center of rotation [32]. This system underwent revisions to reduce complications and enhance implant consistency [32]. In the early 2000s, numerous landmark papers focused on the clinical outcomes of the new Grammont reverse shoulder arthroplasty implant. Three out of the top four publications with the highest number of citations reported the outcomes of the Grammont reverse shoulder arthroplasty implant. The early success of this new system led to its adoption worldwide, resulting in an explosion of shoulder arthroplasty-related publications. In fact, the success of the newer implants expanded the indications for shoulder arthroplasty [9,10]. With the incidence of shoulder arthroplasty increasing every year, it is expected that the number of publications related to shoulder arthroplasty will also follow this upward trend [9,10].

Authors and institutions from the USA significantly contributed to the realm of shoulder arthroplasty research, with up to half (48.1%) of the publications authored by USA researchers and clinicians. Nevertheless, French authors had a higher impact in this field, as five of the top 10 publications with the highest number of citations were authored by French researchers and clinicians. French authors are also heavily involved in multinational collaborations, with up to one-third (33.3%) of their publications being produced in collaboration with others. They had the most collaborative projects with authors from the USA and Switzerland. As discussed previously, this may be attributed to the Grammont-designed reverse shoulder arthroplasty that is being popularized in other parts of the world. Notably, there is a huge vacuum in the developing world in the field of shoulder arthroplasty. This may be due to a combination of the high cost of shoulder arthroplasty implants and the prioritization of other healthcare problems, like infectious diseases, in developing countries [4].

In terms of the breakdown of article types, RTSA-related publications dominated the field of shoulder arthroplasty, accounting for 45.5% of the total publications. The advancement in implant design may be the reason for the high volume of RTSA-related articles. With modern designs, patients who undergo RTSA experience more consistent and better functional outcomes compared to those who undergo hemiarthroplasty or TSA [9,10,32]. Patients with rotator cuff deficiency are better suited for RTSA, as the implant alters the biomechanics of the shoulder, allowing surrounding muscles such as the deltoid to compensate for the loss of rotator cuff muscles [9,10].

Limitations

This bibliometric analysis has several fundamental limitations. Selection bias is present in this review, as only English articles curated from the Scopus database are included. Besides that, the literature search is limited up to the year 2023, potentially underscoring the number of citations received by some of the articles, especially those articles that were published recently. In this bibliometric analysis, all the articles from the time of Scopus database inception to the year 2023 were reviewed and included if they fit the selection criteria. The wide range of publication dates may affect the authors' affiliation and country, as authors may have moved to other institutions or countries. Furthermore, using only the Scopus database may result in variation in citation counts compared to other databases such as Web of Science Core Collection or Google Scholar. 

## Conclusions

With the improvement of the shoulder arthroplasty implant designs, the results of shoulder arthroplasty are increasingly consistent, leading to more research and publications in this field. The turn of the century marks a turning point in shoulder arthroplasty, as newer implant designs revolutionized the field. This bibliometric analysis examines shoulder arthroplasty-related publications through the lens of the Scopus database, offering insight into research trends and collaboration among authors, institutions, and countries. Despite exponential growth in shoulder arthroplasty-related research and publications, this technology is still confined to developed countries. 
